# Retrospective analysis of the use of G-CSF and its impact on dose response for anthracycline plus taxane-based schedules in early breast cancer

**DOI:** 10.1007/s12094-013-1153-7

**Published:** 2014-02-15

**Authors:** J. A. Pérez-Fidalgo, B. Bermejo, I. Chirivella, M. T. Martínez, I. González, J. M. Cejalvo, I. Catoira, P. Martínez, E. Contel, A. Lluch

**Affiliations:** 1 Medical Oncology Service, Hospital Clínico Universitario Avda, Blasco Ibáñez, 17, 46011 Valencia, Spain; 2 Instituto de Investigación Sanitaria INCLIVA, Valencia, Spain

**Keywords:** Anthracyclines, Early breast cancer, Granulocyte colony-stimulating factor, Observational study, Taxanes

## Abstract

**Purpose:**

To evaluate the impact on survival of the relative dose intensity (RDI) achieved in patients with early breast cancer receiving anthracycline plus taxane-based chemotherapy in the adjuvant setting.

**Patients and methods:**

Patients with early breast cancer diagnosed from January 1999 through December 2006 were included. Dose intensity was evaluated according to the number of delayed cycles and days and the percentage of RDI.

**Results:**

A total of 231 breast cancer patients were included. Granulocyte colony-stimulating factor (G-CSF) was given to 39 % of patients. Few patients delayed chemotherapy administration ≥2 cycles (6 %) and ≥15 days (2 %), and the majority of them received ≥85 % of the RDI (98 %). Overall survival was statistically lower at 5 years in patients who received <85 % of RDI in comparison with those who received ≥85 % of RDI (80 vs. 97 %; *p* = 0.026).

**Conclusions:**

With a wide use of G-CSF in patients treated with adjuvant anthracyclines plus taxane-based schedules, 98 % of patients received a RDI ≥85 %. A significant although inconsistent impairment of survival was found in those patients with lower RDI.

## Introduction

Adjuvant chemotherapy has shown increased survival in early breast cancer. About 6 months of adjuvant anthracycline-based polychemotherapy schedules (FAC or FEC) reduced the annual breast cancer death rate by about 38 % for women younger than 50 years and by about 20 % for those diagnosed at 50–69 years of age [1] compared to non-anthracycline-containing schedules.

The benefit of addition of taxanes to anthracycline-based schedules was confirmed in a meta-analysis of thirteen studies including 22,903 patients [2]. The pooled hazard ratio (HR) estimated was 0.83 for disease-free survival (DFS) and 0.85 for overall survival (OS). The risk reduction was not influenced by the type of taxane, by estrogen receptor (ER) expression, by the number of axillary metastases (N1 to 3 vs. N4), or by the patient’s age/menopausal status.

Several trials have studied the relationship between dose intensity and survival. Muss et al. [3] observed in a retrospective study with 6,487 older and younger patients with lymph node-positive breast cancer that the dose level and the dose intensity of chemotherapy were significantly related to OS and DFS, independently of the patient’s age. The observed reduction in the hazard of failure of relapse was 22 % for patients who received more intensive chemotherapy in comparison with those who received less intensive chemotherapy (HR: 0.78; 95 % confidence interval (CI), 0.72–0.85).

In another recent retrospective study reported by our group, the dose response effect was identified as a crucial factor in the administration of anthracycline-based non-taxane schedules for the adjuvant treatment of early breast cancer [4]. Optimal delivery of the programmed chemotherapy improved OS and DFS. Thus, delays and/or reductions of chemotherapy should be avoided whenever possible to achieve the maximal benefit for the patient.

Breast cancer treatment is not exempt from adverse events, with neutropenia and febrile neutropenia being two of the most common and life-threatening side effects of adjuvant chemotherapy. Although the incidence of these adverse events may be minimized with dose reductions and delays in treatment, the most common strategy to maintain dose intensity is to administer granulocyte colony-stimulating factors (G-CSF) during chemotherapy administration [5]. According to the recommendations of the American Society of Clinical Oncology, the use of G-CSF as primary prophylaxis is justified in patients who are at high risk of febrile neutropenia based on age, medical history, disease characteristics, and the myelotoxicity risk of the chemotherapy regimen given. Moreover, it is recognized that G-CSF may allow a modest to moderate increase in the dose intensity of chemotherapy [6].

Studies with dose-dense taxane-containing regimens followed with G-CSF support have shown contradictory results on survival in early breast cancer in different studies [7, 8]. However, little is known about the impact of maintaining relative dose intensity (RDI) in conventional chemotherapy schedules including taxanes.

The aim of our study was to assess the impact on DFS and OS of dose-density of anthracyclines plus taxane-based schedules in the adjuvant setting of patients with early breast cancer. A secondary objective of this study was to evaluate the dose intensity achieved in a non-selected population treated with adjuvant anthracyclines and taxanes in whom G-CSF was administered at clinician’s discretion.

## Patients and methods

### Study design

A retrospective database analysis was performed in January 2010. The database was created in 1980 and, since then, clinical data from all patients with breast cancer treated at the Hospital Clínico Universitario of Valencia (Spain) have been entered from diagnosis to death.

Confidentiality of patients’ data was maintained throughout the study. Data retrieval was performed by two data managers. Four independent medical oncologists verified 15 % of the retrieved data against the original medical records to confirm accuracy. The study was performed according to local legislation and the study protocol was approved by the Ethics Committee of Hospital Clínico Universitario of Valencia (Spain).

### Study procedures

Patients to be included into this retrospective analysis were to have a diagnosis of early breast cancer from January 1999 through December 2006, a surgical procedure as the primary treatment of the disease, and an anthracycline plus taxane-based chemotherapy given in the adjuvant setting.

In the adjuvant setting, patients received anthracyclines plus either docetaxel or paclitaxel in different schedules based on the individual characteristics of the patient and the tumor. Hormonal therapy was started after chemotherapy completion and continued for 5 years in all patients with hormone receptor-positive tumors. Radiotherapy was initiated within approximately 4 weeks after the last cycle of chemotherapy in all patients who had undergone breast-conserving surgery or who had a tumor size >5 cm or ≥4 lymph nodes affected. G-CSF support was administered as primary or secondary prophylaxis, or to manage adverse events at clinician’s discretion.

Data retrieved included patient age, year of diagnosis, tumor stage, histological grade, as well as menopausal and hormonal receptor status. Other treatment-related data were retrieved such as type of surgery and radiotherapy, the type of hormonal therapy given, chemotherapy schedules administered, mean percentage of administered dose throughout the cycles, the number of chemotherapy cycles delayed and the number of delayed days during chemotherapy treatment. Lastly, the final day of follow-up along with any event (disease recurrence or death) that occurred during the follow-up period was also noted. To ensure consistency of data, patients not diagnosed in this hospital were excluded.

### Statistical analysis

The primary objective of this analysis was to evaluate whether the optimal delivery of an anthracycline plus taxane-based schedule in the adjuvant setting of patients with early breast cancer could impact on DFS and OS at 5 years. OS at 5 years was defined as being alive 5 years after cancer diagnosis. Similarly, DFS at 5 years was defined as being alive, with no disease recurrence, 5 years after cancer diagnosis. Secondary objectives included evaluating the dose intensity achieved in a non-selected population treated with anthracyclines and taxanes in whom G-CSF was administered at clinician’s discretion and knowing clinical, epidemiological and treatment characteristics of this population.

Three variables were chosen to assess chemotherapy delivery to the patient, which were the number of delayed cycles, the number of delayed days, and the percentage of RDI given. The number of delayed cycles was based on whether the patient had more than two cycles with ≥3 days of delay with respect to the planned schedule (<2 delayed cycles, ≥2 delayed cycles). The number of delayed days during treatment administration was based on whether or not the patient’s chemotherapy had to be delayed more than 14 days overall (<15 delayed days, ≥15 delayed days). Finally, the RDI was based on whether or not the patient’s RDI was less than 85 % (≥85 %, <85 %). RDI was calculated as the mean percentage of administered dose throughout the entire treatment multiplied by the ratio of the number of treatment days as planned to the number of treatment days as planned plus the number of delayed days. DFS and OS at different time points were defined as the time from cancer diagnosis until the occurrence of the event (disease recurrence or death).

Collected variables in the case report form were presented with absolute and relative frequencies for qualitative variables, and with measures of association and dispersion for quantitative variables (mean, standard deviation, median, minimum and maximum). Times to event variables were described using the Kaplan–Meier method. Possible relationships between qualitative variables were studied by Cox regression. All statistical tests were performed against a two-sided, alternative hypothesis using a significance level of 0.05 and a 95 % CI. The SPSS (version 17.0) statistical program (SPSS Inc., Chicago, IL, USA) was used for the statistical analysis.

## Results

### Patient’s characteristics at diagnosis

A total of 231 patients with early breast cancer diagnosed from 1999 to 2007 in our institution were included in this analysis. Patients’ characteristics at diagnosis are shown in Table 1.

**Table 1:** Patient characteristics at diagnosis (*n* = 231)

Characteristics	*n*	%
Age (years), *n* = 231		
Median (range)	59 (26–80)	
Treatment period, *n* = 231		
1999–2002	100	43
2002–2007	131	57
Tumor stage, *n* = 231		
I	87	38
II	22	9
IIIA	120	52
IIIB	2	1
Histological grade, *n* = 225		
I	37	17
II	118	52
III	70	31
Lymph node involvement, *n* = 231		
0	98	42
1–3	99	43
4–9	27	12
≥10	7	3
Receptor expression status		
ER+, *n* = 229	176	77
PR+, *n* = 226	147	65
ER+ or PR+, *n* = 226	187	83
HER2+, *n* = 194	96	49
Triple negative (ER−, PR−, HER2−), *n* = 192	14	7
Menopausal status, *n* = 230		
Pre-/perimenopausal	101	44
Postmenopausal	129	56
Hormonal treatment and related agents, *n* = 231		
Yes	188	81

*ER* estrogen receptor, *PR* progesterone receptor, *HER2* human epidermal growth factor receptor 2

### Treatment administration in the adjuvant setting

All patients underwent surgery, either conservative (62 %) or mastectomy (38 %). Radiotherapy was given to 70 % of patients, and 81 % of them received hormonal therapies such as tamoxifen (72 % of patients), anastrozole (31 %), exemestane (15 %) and letrozole (15 %). All patients received adjuvant chemotherapy with anthracyclines and taxanes, of whom 68 % included docetaxel and 32 % included paclitaxel. The chemotherapy schedule most frequently administered was FAC × 4 → T × 8 (21 %) followed by TAC × 6 (20 %). Other treatment schedules given are described in Table 2.

**Table 2:** Characteristics of the treatments administered to the patients

Adjuvant chemotherapy (cycles), *n* = 231	*n*	%
With docetaxel	**157**	**68**
TAC × 6	46	20
ET × 4 → X × 4	39	17
EC × 4 → T × 4	39	17
AT × 4 → CMF × 4	21	9
A × 3 → T × 3 → CMF × 3	12	5
With paclitaxel	**74**	**32**
FAC × 4 → T × 8	48	21
AT × 4 → CMF × 3	13	5
FEC × 4 → T × 8	11	5
AC × 4 → T × 8 or T × 12	2	1

Chemotherapy administration, *n* = 231	*n*	%
Delayed cycles		
<2 cycles	216	94
≥2 cycles	15	6
Delayed days		
<15 days	226	98
≥15 days	5	2
Relative dose intensity		
≥85 %	226	98
<85 %	5	2

Prophylaxis with G-CSF, *n* = 231	*n*	%
Yes	**84**	**36**
Primary prophylaxis	55	24
Secondary prophylaxis	29	12
No	**147**	**64**
G-CSF given later to treat neutropenia	8	3

*A* doxorubicin, *C* cyclophosphamide, *E* epirubicin, *F* 5-fluorouracil, *G-CSF* granulocyte colony-stimulating factor, *M* methotrexate, *T* taxane (docetaxel or paclitaxel), *X* capecitabine

At clinician’s discretion, G-CSF was given as primary or secondary prophylaxis to 24 and 12 % of patients, respectively. Additionally, 3 % of patients received G-CSF later on to manage toxicity. G-CSF was mostly given to patients treated with docetaxel-based schedules, either as primary (98 %) or secondary prophylaxis (86 %). Only five patients treated with paclitaxel and anthracyclines received G-CSF, four as secondary prophylaxis (Table 3). Clinicians mostly decided to give G-CSF as primary prophylaxis in patients receiving TAC schedule (78 %), followed by those treated ET → X (46 %). However, in patients treated with EC → T, G-CSF was not given as primary prophylaxis but had to be introduced as secondary prophylaxis in 44 % of patients. None of the patients treated with AT → CMF or A → T → CMF received any type of prophylaxis with G-CSF. Among patients receiving paclitaxel-based schedules, only one patient treated with FAC → T received primary prophylaxis with G-CSF, and in four additional patients G-CSF was introduced later on.

**Table 3:** Administration of prophylaxis with G-CSF according to investigator’s criteria

Treatment	Prophylaxis with G-CSF		
	No prophylaxis, *n* (%)	Primary, *n* (%)	Secondary, *n* (%)
With docetaxel			
TAC × 6, *n* = 46	7 (15)	36 (78)	3 (7)
ET × 4 → X × 4, *n* = 39	16 (41)	18 (46)	5 (13)
EC × 4 → T × 4, *n* = 39	22 (56)	0 (0)	17 (44)
AT × 4 → CMF × 4, *n* = 21	21 (100)	0 (0)	0 (0)
A × 3 → T × 3 → CMF × 3, *n* = 12	12 (100)	0 (0)	0 (0)
With paclitaxel			
FAC × 4 → T × 8, *n* = 48	43 (90)	1 (2)	4 (8)
AT × 4 → CMF × 3, *n* = 13	13 (100)	0 (0)	0 (0)
FEC × 4 → T × 8, *n* = 11	11 (100)	0 (0)	0 (0)
AC × 4 → T × 8 or T × 12, *n* = 2	2 (100)	0 (0)	0 (0)
Total	147 (64)	55 (24)	29 (12)

*A* doxorubicin, *C* cyclophosphamide, *CI* confidence interval, *E* epirubicin, *F* 5-fluorouracil, *G-CSF* granulocyte colony-stimulating factor, *M* methotrexate, *T* taxane (docetaxel or paclitaxel), *X* capecitabine

Overall, few patients had to delay and/or reduce dosages during the administration of adjuvant chemotherapy. Five patients (2 %) received less than 85 % of RDI, 15 patients (6 %) delayed two or more treatment cycles, and five patients (2 %) had a delay of 15 days or more. Severe neutropenia and febrile neutropenia were present in 42 (18 %) and 23 patients (10 %), respectively.

### Impact of adjuvant chemotherapy on DFS and OS

The probability of surviving without recurrence of the disease at 5 and 10 years was 88 % (95 % CI 84–92) and 80 % (95 % CI 72–87), respectively; and the probability of being alive, with or without disease recurrence, was 97 % (95 % CI 95–99) and 82 % (95 % CI 72–92), respectively (Tables 4, 5).

**Table 4:** Impact of chemotherapy delivery on disease-free survival

DFS	Survival probability (%)						
	<2 cycles (*n* = 216)	≥2 cycles (*n* = 15)	<15 days (*n* = 226)	≥15 days (*n* = 5)	≥85 % RDI (*n* = 226)	<85 % RDI (*n* = 5)	Overall % (95 % CI)
2 years	99	93	98	100	99	80	98 (97–100)
*p*	0.126		0.765		0.001*		
4 years	91	93	91	100	91	80	91 (87–95)
*p*	0.791		0.491		0.315		
5 years	88	93	88	100	88	80	88 (84–92)
*p*	0.562		0.418		0.500		
6 years	86	93	86	100	87	80	87 (82–91)
*p*	0.473		0.375		0.563		
8 years	84	93	85	100	85	80	84 (79–90)
*p*	0.465		0.366		0.621		
10 years	79	93	79	100	80	80	80 (72–87)
*p*	0.418		0.323		0.695		

*CI* confidence interval, *DFS* disease-free survival, *RDI* relative dose intensity

* Statistically significant

**Table 5:** Impact of chemotherapy delivery on overall survival

OS	Survival probability (%)						
	<2 cycles (*n* = 216)	≥2 cycles (*n* = 15)	<15 days (*n* = 226)	≥15 days (*n* = 5)	≥85 % RDI (*n* = 226)	<85 % RDI (*n* = 5)	Overall % (95 % CI)
2 years	99	93	99	100	99	100	99 (97–100)
*p*	0.061		0.796		0.796		
4 years	98	93	97	100	98	80	97 (95–99)
*p*	0.302		0.712		0.014*		
5 years	97	93	97	100	97	80	97 (95–99)
*p*	0.395		0.687		0.026*		
6 years	93	93	93	100	93	80	93 (89–97)
*p*	0.849		0.539		0.135		
8 years	92	93	92	100	92	80	92 (87–96)
*p*	0.902		0.514		0.165		
10 years	81	93	81	100	82	80	82 (72–92)
*p*	0.888		0.418		0.245		

*CI* confidence interval, *RDI* relative dose intensity, *OS* overall survival

* Statistically significant

**Fig. 1:**
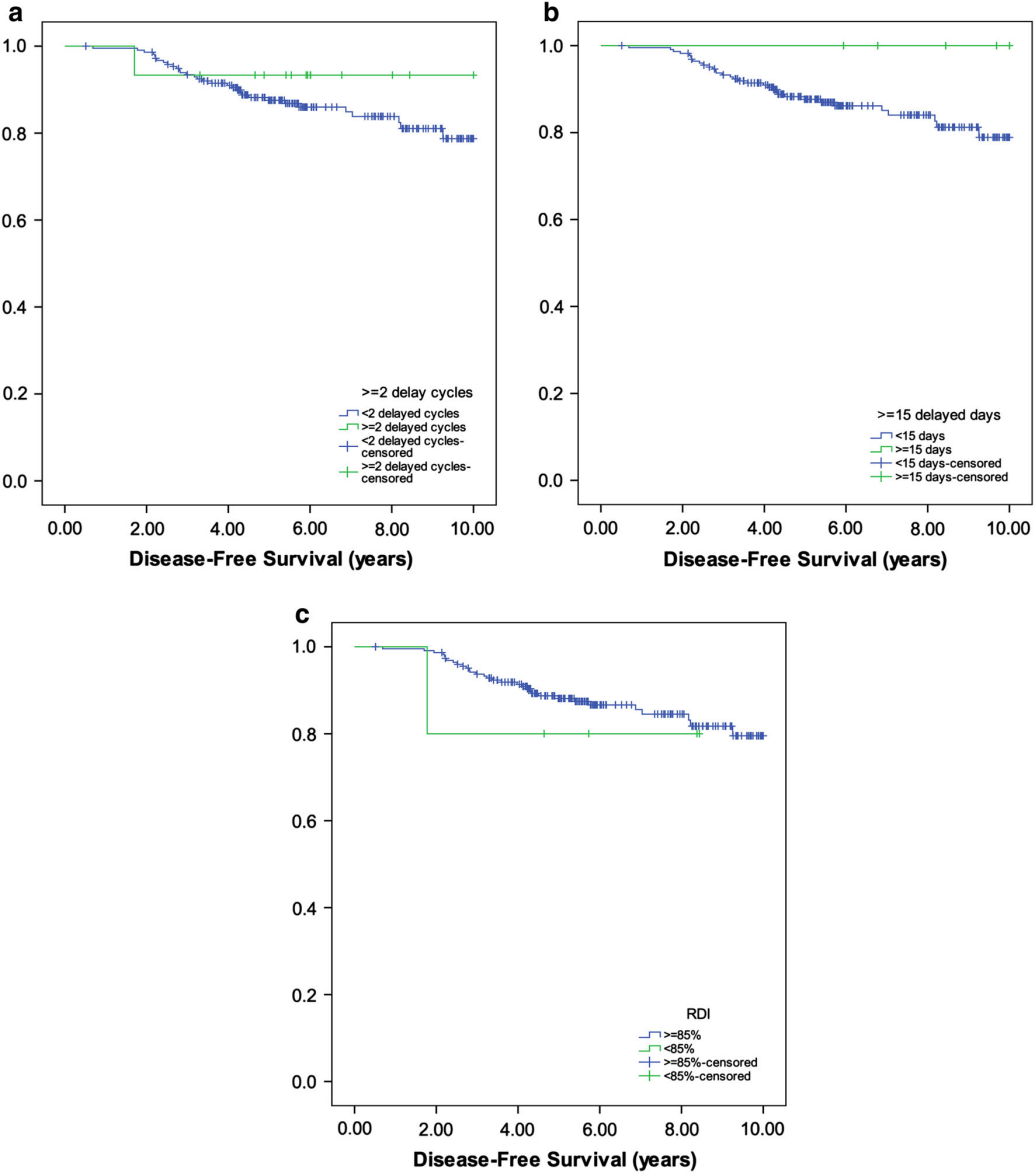
**a** Number of delayed cycles (<2 cycles, ≥2 cycles), **b** number of delayed days (<15, ≥15 days), **c** RDI (≥85, <85 %)

**Fig. 2:**
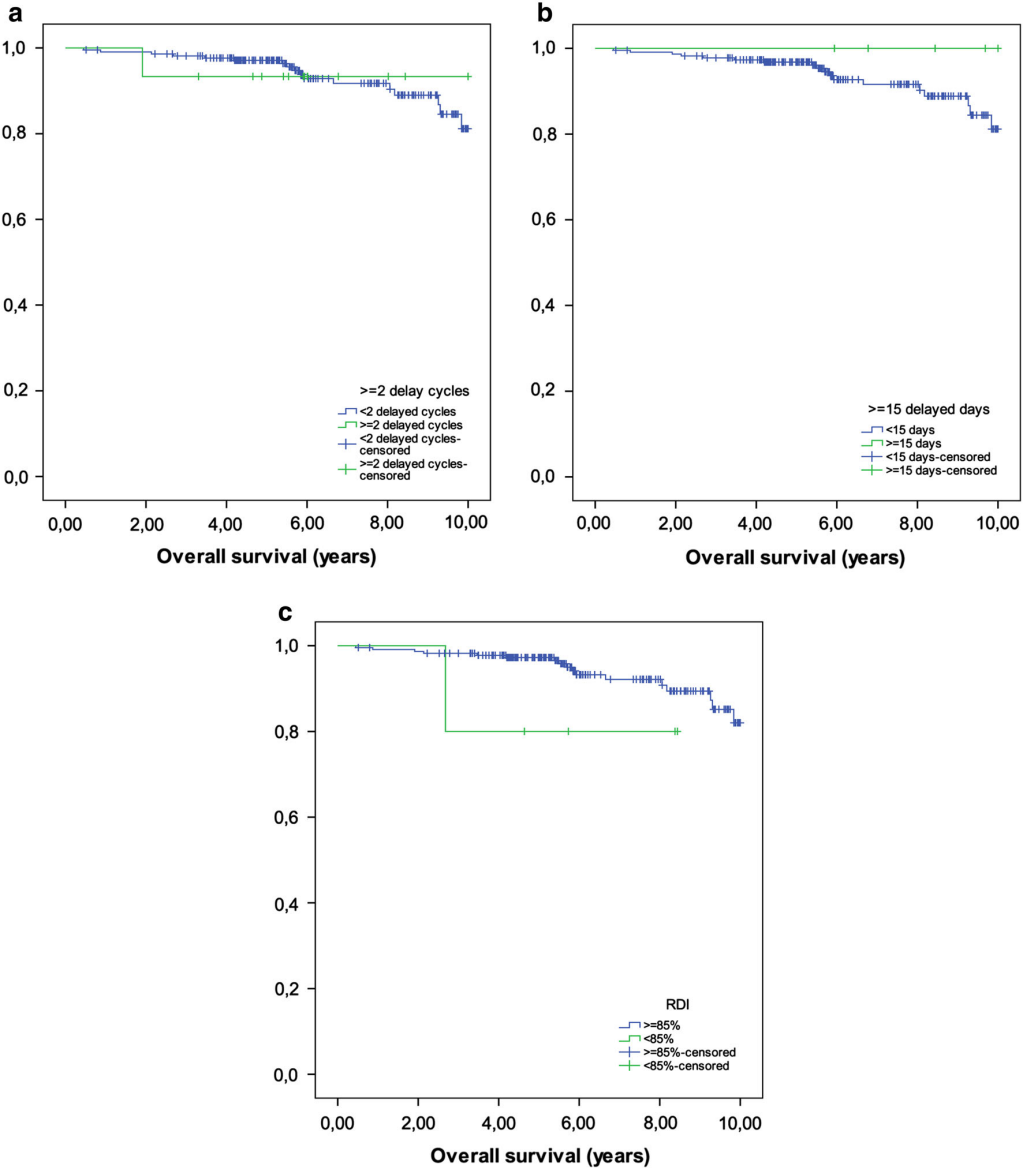
**a** Number of delayed cycles (<2 cycles, ≥2 cycles), **b** number of delayed days (<15, ≥15 days), **c** RDI (≥85, <85 %)

As shown in Tables 4 and 5 and Figs. 1 and 2, DFS and OS were not affected by the number of delayed cycles and the number of delayed days. However, patients who received a reduced RDI (<85 %) had significantly lower probability of survival without recurrence of disease at 2 years in comparison with patients who received ≥85 % (80 vs. 99 %; *p* = 0.001), and a lower probability of being alive at 4 and 5 years (98 vs. 80 %; *p* = 0.014; 97 vs. 80 %; *p* = 0.026, respectively).

## Discussion

Our results showed that DFS and OS were not affected by delays in scheduled chemotherapy. Only those patients who received a reduced RDI had a lower probability of survival without recurrence of disease at 2 years. Also, patients who received reduced RDI had a lower probability of being alive at 4 and 5 years. However, the impact on DFS and OS is not consistent along years.

Low RDI is a common issue in clinical practice. In a population study of 1,243 community oncology practice in the USA with data from 20,799 early breast cancer patients showed that 36.5 % presented dose reductions of ≥15 days and 24.9 % had treatment delays ≥7 days resulting in 55.5 % of patients receiving RDI <85 %. Multivariate analysis identified the absence of primary prophylaxis with G-CSF as one of the independent predictors for low RDI [9]. A later study by the same group including 2,280 women with early breast cancer from community practice, low dose intensity in conventional schedules was reduced, probably due to the introduction of G-CSF, but the frequency of inappropriate chemotherapy delivery was 31, 24 and 26 % for dose delays, dose reductions and low RDI, respectively [10]. Strikingly, in our series the proportion of patients with low RDI was very small (2 %). This is probably as a consequence of a wide use of prophylactic G-CSF in our practice. This difference in sample size between patients with normal versus low RDI is the main limitation of our study and this issue precludes definitive conclusions.

In 2009, our group, Chirivella et al. [4] assessed how the suboptimal delivery of an anthracycline-based non-taxane adjuvant chemotherapy may impact the outcome of patients with early breast cancer. Delays of >2 cycles, ≥15 delayed days or RDI <85 % had a statistical impact on DFS. Moreover, when clinically relevant disease characteristics were controlled, such as the number of lymph nodes affected and the hormonal receptor status, the significance on DFS remained. However, in contrast with our study, prophylactic G-CSF was not widely used, as a result, the percentage of patients who had delayed chemotherapy administration or who received a reduced dose of chemotherapy was much higher than in our series. Patients who delayed chemotherapy >2 cycles or ≥15 days were 27 and 29 %, respectively, and 12 % received <85 % of RDI. In the current analysis, in which 36 % of patients received G-CSF and primary or secondary prophylaxis at clinician’s discretion, these percentages went down to 6, 2 and 2 %, respectively. Likely as a result of this, the impact observed in the outcome of patients in terms of DFS and OS was also smaller.

The role of G-CSF on maintaining an appropriate RDI in taxanes-containing schedules for breast cancer patients has been previously analyzed. Martin et al. [11] assessed the toxicity and the health-related quality of life of patients with breast cancer treated with anthracyclines, with or without taxanes, and with or without primary prophylactic G-CSF. In the group of patients treated with taxanes, 96 % of patients who received prophylaxis with G-CSF completed the six-cycle schedule, in comparison with 90 % of patients without G-CSF prophylaxis (*p* = 0.019). In comparison with patients who received secondary prophylaxis with G-CSF, primary prophylaxis was associated with a significant reduction in the number of cases of febrile neutropenia (26 vs. 7 %; *p* < 0.001) and grade 2/3 anemia (47 vs. 28 %; *p* < 0.001); and fewer patients required red cell transfusions (7 vs. 2 %; *p* < 0.010). Primary prophylaxis with G-CSF was also associated with a significant reduction in the incidence of asthenia, anorexia, myalgia, nail disorders and stomatitis compared with secondary prophylactic G-CSF. The reduction of these adverse events would facilitate compliance of the treatment as we have observed in our study.

It has been demonstrated that TAC schedules are more toxic than FAC schedules, not only with respect to neutropenic fever events, but also with respect to many extrahematological side-effects such as asthenia, stomatitis, diarrhea and myalgia [12]. In our study, those patients who received more aggressive treatment schedules, primary prophylaxis with G-CSF has been widely used in highly toxic regimens such as TAC. Overall, 78 % of patients treated with TAC and 46 % of patients treated with ET → X received primary prophylaxis with G-CSF. However, when less toxic schedules were given, only 2 % received primary prophylaxis and 8 % received secondary prophylaxis. Interestingly, none of the patients treated with EC → T received G-CSF as primary prophylaxis, but in 44 % of them it had to be introduced later on as secondary prophylaxis. Hence, it may be more beneficial for certain patients treated with EC → T to be given G-CSF from the beginning to avoid the occurrence of adverse events later on.

According to clinical guidelines, use of G-CSF is recommended to maintain chemotherapy if reduction of dose-density is associated to poor prognosis or if the risk of febrile neutropenia is high (≥20 %) [13]. The important rate of prophylactic G-CSF administration in our study, although in accordance to clinical guidelines, may be a relevant factor to explain the high rate of RDI >85 % observed in our study.

Strikingly, a Cochrane Database Systematic Review including eight randomized clinical trials assessing the effect of prophylactic G-CSF showed evidence of prevention of febrile neutropenia but failed to confirm any effect on maintaining dose density in breast cancer patients [14]. However, in most of the trials analyzed the chemotherapy regimens used had a risk of febrile neutropenia that was below the threshold at which current guidelines recommend routine primary prophylaxis. Moreover the small number of evaluable patients in some trials and the variability of definitions may strongly bias these findings.

Of note, at 5 and 10 years, the rate of DFS in our study was 88 and 80 %, respectively; and the rate of OS was 97 and 82 %, respectively. These data are outstanding taking into account, that more than a half of our patients were diagnosed with stage III breast cancer. Although it is impossible to identify which factors were responsible for these results, it seems reasonable to suppose that both the addition of taxanes to an anthracycline-based chemotherapy in the adjuvant setting, together with the optimal administration of chemotherapy, may have played an important role.

In fact, it cannot be discarded that reduced survival in many treatment schedules in the adjuvant setting may be due, at least in part, to a reduced compliance of the RDI [15]. Also, the high frequencies of adverse events that occur as a result of more aggressive therapies further hinder compliance with therapy. In this respect, the administration of prophylactic G-CSF at clinician’s discretion in those patients receiving more aggressive schedules could help to improve this compliance. On the other hand, it is important to note that nodes positivity and hormone receptors are key variables related with outcome in patients with breast cancer. However, in our study the number of patients with RDI <85 % is too small to conduct a multivariate analysis on this relevant issue.

In summary, our results are inconclusive for the primary endpoint. However, despite the previously mentioned limitations, our results suggest that, with the adequate use of G-CSF in patients with breast cancer treated with anthracyclines plus taxane-based schedules in the adjuvant setting, optimal chemotherapy administration could be achieved in almost all patients.
